# Quantifying individual specialization using tracking data: a case study on two species of albatrosses

**DOI:** 10.1007/s00227-018-3408-x

**Published:** 2018-09-08

**Authors:** A.-S. Bonnet-Lebrun, R. A. Phillips, A. Manica, A. S. L. Rodrigues

**Affiliations:** 1 Department of Zoology, University of Cambridge, Downing Street, Cambridge, CB2 3EJ UK; 2 CEFE, UMR 5175, CNRS, Université de Montpellier, Université Paul-Valéry Montpellier, EPHE, 34293 Montpellier, France; 3 British Antarctic Survey, Natural Environment Research Council, Madingley Road, High Cross, Cambridge, CB3 0ET UK

## Abstract

Many predictive models of spatial and temporal distribution (e.g. in response to climate change or species introductions) assume that species have one environmental niche that applies to all individuals. However, there is growing evidence that individuals can have environmental preferences that are narrower than the species niche. Such individual specialization has mainly been studied in terms of dietary niches, but a recent increase in the availability of individual movement data opens the possibility of extending these analyses to specialisation in environmental preferences. Yet, no study to date on individual specialisation has considered the environmental niche in its multidimensionality. Here we propose a new method for quantifying individual specialisation in multiple dimensions simultaneously. We compare the hypervolumes in *n*-dimensional environmental niche space of each individual against that of the population, testing for significant differences against a null model. The same method can be applied to a 2-dimensional geographic space to test for site fidelity. We applied this method to test for individual environmental specialisation (across three dimensions: sea surface temperature, eddy kinetic energy, depth) and for site fidelity among satellite-tracked black-browed albatrosses (*Thalassarche melanophris*) and grey-headed albatrosses (*Thalassarche chrysostoma*), during chick-rearing at South Georgia. We found evidence for site fidelity in both species and of environmental specialisation among individual grey-headed but not black-browed albatrosses. Specialisation can affect the resilience of populations affected by natural and anthropogenic changes in the environment, and hence has implications for population dynamics and conservation.

**Electronic supplementary material:**

The online version of this article (10.1007/s00227-018-3408-x) contains supplementary material, which is available to authorized users.

## Introduction

The spatial distribution of a species is influenced by a range of environmental variables which reflect the niche, classically referred to as an *n*-dimensional hypervolume in environmental space (Hutchinson 1957). Aspects of the environment can be categorised as resources or as conditions (Peterson 2011): the former are linked dynamically to the population under study (e.g. resources that are consumed, which can be studied directly or indirectly), whereas the latter are not consumed and not subject to competition (scenopoetic variables; e.g. environmental conditions such as temperature or depth; Hutchinson 1978). Understanding the relationships between species and their environment allows the development of predictive models of species distributions in space or time (Elith and Leathwick 2009; Wakefield et al. 2011; Scales et al. 2016b), including in response to climate change or to species introductions (Elith et al. 2010; Gallardo and Aldridge 2015; Vicente et al. 2016). These models assume a common niche for the whole species or population, but in reality, this niche is the combination of individual preferences or tolerances. Whilst in principle all individuals can have the same broad niche as the species (i.e. generalist individuals), there is often some specialisation at the individual level (Bolnick et al. 2003). Accordingly, there is a growing awareness of the importance of considering intra-specific variation in niches, and in the development of appropriate methodology (Bolnick et al. 2011; Carneiro et al. 2017; Phillips et al. 2017).

Quantifying individual specialisation requires observations of repeated choices made by each individual. This is relatively straightforward for prey selection; numerous studies have shown evidence of individual diet specialisation in a wide range of taxa (Bolnick et al. 2003; Araújo et al. 2011; Phillips et al. 2017 for reviews). However, extending the study of individual specialisation to foraging site and environmental preferences is more complicated: if individuals are sedentary, it is impossible to know whether their environmental preferences encompass broader conditions elsewhere, as only one choice of environment is observed. Species that are very mobile, on the other hand, provide good study models for quantifying environmental specialisation, as animals undertaking long-distance movements can potentially sample a wide range of environmental conditions and make a series of choices (i.e. where to travel next). Data on individual movements are increasingly available because of recent developments in tracking devices, including improved accuracy and miniaturisation (Wakefield et al. 2009a; Ropert-Coudert et al. 2009; Bridge et al. 2011).

In practice, analyses of movement in the natural environment can provide information on individual consistency (i.e., whether individuals repeat their choices in geographical or environmental space) but not directly on individual specialisation (i.e., whether individuals have a narrow ecological niche). Indeed, consistency is a prerequisite but not conclusive evidence for specialisation, as consistency may derive from geographical preferences (e.g. because of memory-based processes) rather than ecological or physiological constraints. The latter can only be tested under laboratory conditions (e.g., testing physiological tolerance to temperature; Bernardo and Spotila 2006), which for many organisms is neither practical nor ethical. Consistency is thus the best available proxy measure for specialisation under natural conditions. For simplicity, throughout we use the term ‘individual specialisation’ rather than ‘individual consistency’, while acknowledging that movement studies are in reality measuring the latter.

Previous studies on individual specialisation in wide-ranging species have focused on differences in behaviour (e.g. timing of migration; Phillips et al. 2005; McFarlane Tranquilla et al. 2014, diving strategies; Report-Coudert et al. 2003; Patrick et al. 2014), or geographical specialisation (also termed ‘site fidelity’; e.g. to migration route or wintering area; Phillips et al. 2005; Dias et al. 2010, or foraging site during the breeding season; Patrick and Weimerskirch 2014; Wakefield et al. 2015). Fewer studies have investigated environmental specialisation (Phillips et al. 2017), but those often find evidence of consistency in the use of certain habitat types or environmental conditions (Phillips et al. 2009a; Catry et al. 2014; Wakefield et al. 2015; Fodrie et al. 2015).

The paucity of studies on environmental specialisation is partly due to methodological limitations. Quantifying environmental specialisation requires a comparison of the environments utilised by individuals across a series of repeated choices of locations (typically breeding or foraging areas) with those utilised by the population as a whole (see Carneiro et al. 2017 for a review of existing methods). When environmental space can be classified into discrete habitat units (e.g. Catry et al. 2014; Fodrie et al. 2015), traditional methods developed for examining consistency in prey choice can be applied (see Bolnick et al. 2002). However, in many cases, no discrete environmental classification is possible without a loss of information and the use of arbitrary thresholds. Preference distributions can be estimated for each variable (reflecting the proportion of time spent in locations characterized by each value of the variable corrected for its availability), for each independent choice of environmental conditions (e.g. for each breeding season or for each foraging trip). The similarity in preference between each independent choice of conditions (e.g. using Bhattacharyya’s affinity) reflects the degree of individual consistency (Wakefield et al. 2015). However, because of the need to calculate Gaussian kernels in several dimensions simultaneously, this approach currently only allows one or two variables to be analysed at a time. This is a key limitation, because the niche is usually better described by more dimensions, especially if there are interactions. There is therefore a pressing need for an approach that quantifies individual specialisation in more dimensions simultaneously.

Here we quantify individual specialisation in two seabird species in which we expect the level to differ, given known differences in their ecology. Seabirds are particularly suited for the study of individual environmental specialisation, as they can move long distances and are central-place foragers during the breeding season (i.e. the accessibility of different environmental conditions is similar for all individuals in a given colony at the same breeding stage). Seabirds can also be tracked with relative ease, particularly the larger species that can be fitted with devices with long battery lives, and detailed information on their environment can be accessed through satellite remote-sensing (Wakefield et al. 2009). Furthermore, as most seabirds breed in dense colonies, divergent foraging strategies might be expected if these reduce intraspecific competition (Bolnick et al. 2011; Evans et al. 2015). Finally, as many seabirds are threatened by human activities, it is important to understand the degree of specialisation in foraging strategies within populations, which will influence the potential for adaptation to environmental change, as well as decisions relating to monitoring and conservation priorities (Phillips et al. 2017).

We focus here on the grey-headed (*Thalassarche chrysostoma*) and black-browed (*Thalassarche melanophris*) albatross. A degree of non-breeding site fidelity and individual consistency in timing of migration has been observed in grey-headed albatrosses (Croxall et al. 2005), and for black-browed albatrosses, individual behavioural differences have been detected in various traits, including at-sea activity patterns (Mackley et al. 2010), trip duration and maximum range during chick-rearing (Patrick and Weimerskirch 2014), and site fidelity during the non-breeding season (Phillips et al. 2005). Individual habitat specialisation has also been detected in black-browed albatrosses during the breeding season (Patrick and Weimerskirch 2014, 2017) but using habitat categories (shelf vs. shelf edge vs. oceanic waters; Patrick and Weimerskirch 2014) or only one environmental variable (bathymetry; Patrick and Weimerskirch 2017), thus ignoring the multidimensionality of the niche. In contrast, Granadeiro et al. (2014) found no evidence of individual specialisation in this species in diet or carbon source (a proxy for habitat) using stable isotope ratios. Whereas some of these studies suggest there may be a degree of individual specialisation (in behaviour, foraging locations or habitat), so far none has tested whether this translates into differences in the multidimensional environmental niche.

To account for preferences in multiple environmental dimensions, we develop a new method, the Multidimensional Individual Specialisation Index (MISI), which relies on hypervolumes of geographical or environmental space use. We detail the rationale for the method as well as the details of its implementation, and how it can be used in a statistical test of individual specialisation. We then apply it to extensive tracking data from the two albatross species, quantifying and testing for both individual site fidelity (in two dimensions of geographic space) and environmental specialisation (in three dimensions in environmental space). We expect different levels of individual specialisation for the two species, which differ in foraging behaviour and distribution. Black-browed albatrosses forage to a greater extent in neritic and shelf-slope waters (Wakefield et al. 2011) where prey availability is expected to be more stable and predictable because small-scale differences in bottom topography entrain krill swarms or fish shoals (Murphy et al. 1997; Duhamel and Hautecoeur 2009). In contrast, grey-headed albatrosses tend to forage mainly in oceanic environments (particularly the Antarctic Polar Front; Xavier et al. 2003a, b; Clay et al. 2016) where prey aggregations are expected to be more unpredictable, and on the edges of dynamic eddies and frontal systems (Silk et al. 2016).

## Methods

### The multidimensional individual specialisation index (MISI)

#### Rationale

The most common approach used to estimate individual specialisation is based on comparing the within-individual and between-individual variances (Nakagawa and Schielzeth 2010; Bolnick et al. 2003; see Carneiro et al. 2017 for a review of methods). When interpreting the results, one should however keep in mind that although the within-individual variation is usually assumed to be directly linked to the level of individual specialisation, it can also encompass other sources of variation (measurement error, differences between biologically relevant categories (e.g. sex), random residual variation or organisms misinterpreting cues and using environments that do not correspond to their preferences (Westneat al. 2015).

**Fig. 1:**
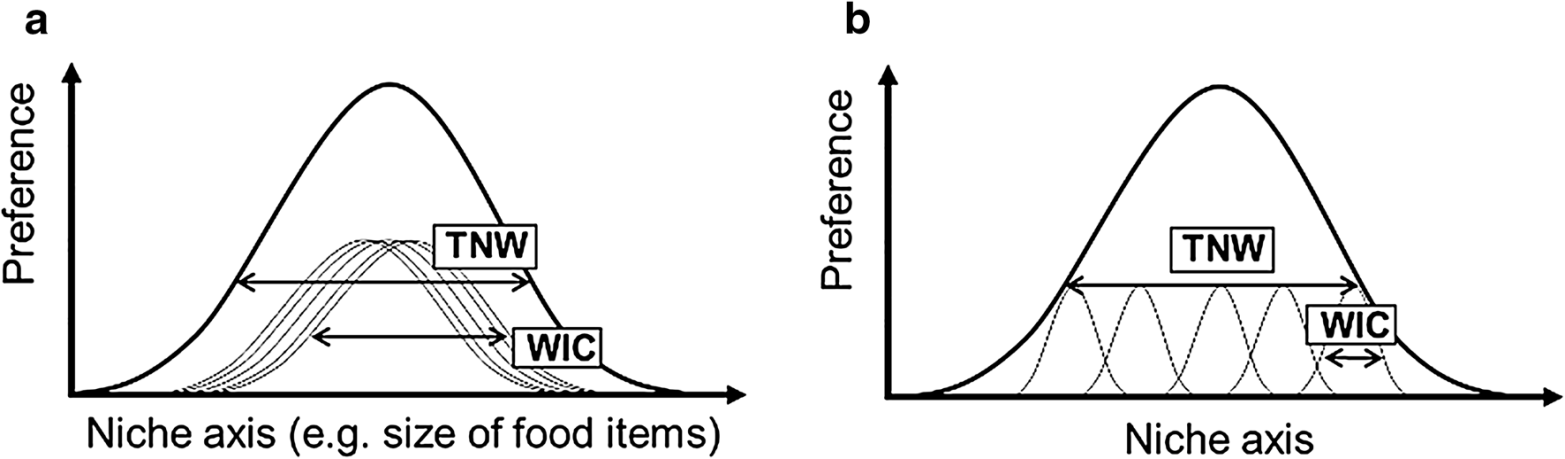
A schematic diagram of how individuals (thin lines) can subdivide the population’s niche (thick line) (adapted from Bolnick et al. 2003). *TNV* total niche volume, *WIC* within-individual component: **a** a population of generalist individuals, **b** a population of specialist individuals

The *MISI* is a generalisation of the approach used in Bolnick et al. (2003) for diet data. For a single continuous variable describing dietary items (e.g. prey size), Bolnick et al. (2003) define the total population niche width (TNW) as the variance in the values of this variable pooled over all consumed items. TNW can be partitioned into a between-individual component and a within-individual component (WIC, the average variance of resources in individual diets; Fig. 1). The ratio $\frac{{\text{WIC}}}{{\text{TNW}}}$ provides information on the level of individual generalism (and 1–$\frac{{\text{WIC}}}{{\text{TNW}}}$ on individual specialisation) within the population.

We extended this approach to the study of environmental preferences. For this we used tracking data consisting of multiple trips per individual. Each trip is a series of geographic locations (*X*_*n*_, *Y*_*n*_), where *n* is the track length and environmental conditions are extracted at each (*X*_*i*_, *Y*_*i*_). Each individual is then characterised by a set of points in as many dimensions as the number of environmental variables considered. Note that the method requires covariate data for each point. The within-individual component for each individual (WIC_*i*_) is calculated as its hypervolume, and the total population niche volume (TNV) as the hypervolume enclosing all the locations visited by all the individuals in the study. Note that, contrary to Bolnick et al. (2003), here we calculate niche volumes and not niche widths, because preferences are characterised in more than one dimension. The Multidimensional Individual Specialisation Index is defined as:

$${\text{MISI}}_{i} = 1 - \frac{{{\text{WIC}}_{i} }}{\text{TNV}}.$$

Note that in the original approach in Bolnick et al. 2003, there is only one average WIC value calculated for the whole population. Although here we use median population values (see Test of individual specialisation), our extension calculates a value for each individual, which takes into account the points raised by Cleasby et al. (2015), that there can be between-individual differences in within-individual variation.

#### Hypervolume construction

**Fig. 2:**
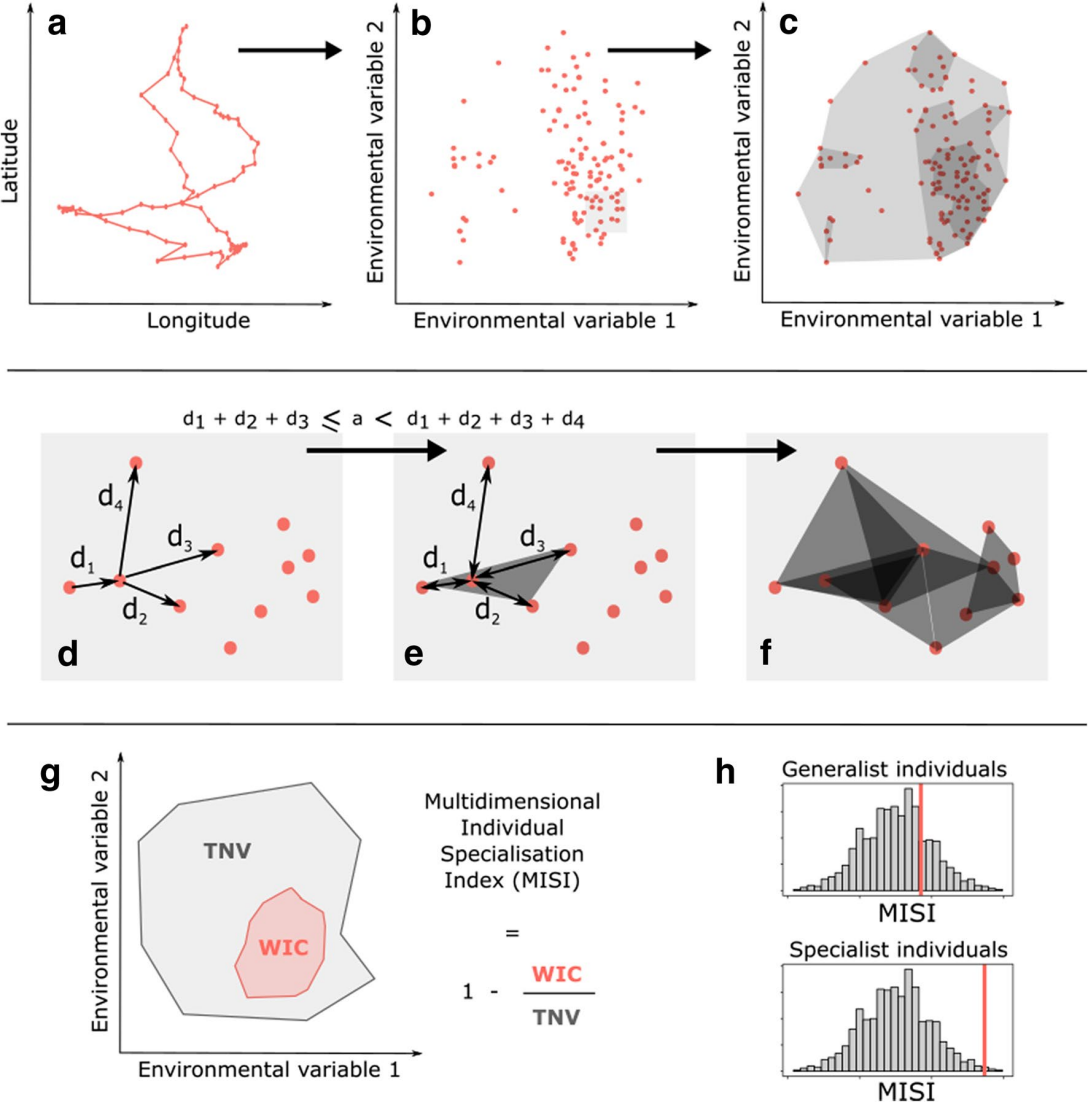
Steps for calculating the Multidimensional Individual Specialisation Index (MISI), illustrated here in two dimensions. **a**: Hypothetical track in geographical space: each point corresponds to a location record. **b** Position of location records in a two dimensional environmental space. **c** Hypervolumes around the locations in environmental space. **d**–**e** Selection of neighbours around a focal point. Neighbours are included from the closest to the furthest, until the sum of the distances between the focal point and its neighbours reaches the value a. Creation of the hypervolume containing all these selected points. **f** Repetition of the previous step for each focal point. **g** Calculation of the index based on the volumes of the individual hypervolume (*WIC* within-individual component) and of the population hypervolume (*TNV* total niche volume). **h** Test of individual specialisation: null distribution of individual specialisation values, grey: 95% CI, red: median of empirical MISI values. If the empirical MISI values fall within the 95% CI of the null distribution, individuals are considered no more specialised than expected by chance. If the empirical MISI values are higher than the 0.975 quantile of the null distribution, individuals are considered more specialised than expected by chance

In order to estimate niche widths, hypervolumes of use are built in niche space (environmental space) by extending a method previously developed for two-dimensional geographical space: the Local Convex Hull (LoCoH) method (Getz et al. 2007). Among the three versions of the LoCoH method, we focused on the so-called adaptive-LoCoH. For each focal point, we first find the maximum number of nearest neighbours such that the cumulative distance between the focal point and its neighbours is less than or equal to a threshold parameter *a*. Note that we take *a* to be the same for all individuals. We then build the smallest convex polyhedron containing these points (Fig. 2c–f); polyhedra are thus smaller where the density of points is higher. To build a polyhedron in *n* dimensions, a minimum of *n *+ *1* points is needed; points with less than *n *+ 1 neighbours satisfying the distance criterion are ignored, providing a filter for outliers. For a given individual, all valid polyhedra are then merged together to obtain the *n*-dimensional niche hypervolume.

Combining n-dimensional polyhedra is mathematically and computationally challenging. We therefore calculate the overall volume of each individual’s niche hypervolume by first intersecting all the individual polyhedras with a multidimensional grid (i.e. checking which cell centroids are included in each polyhedron), and then computing the overall volume as the number of cell centroids that are included in at least one of the constituting polyhedra. The same procedure is used for estimating the population volume, calculated as the number of cell centroids that are included in at least one of all polyhedra of all individuals. To decide the appropriate grain size for the grid, the volumes of the polyhedra are calculated for decreasing grain sizes until MISI values stabilise. We developed a simple implementation of this algorithm in R programming language that can deal efficiently with up to four dimensions (available in Supplementary Materials).

The construction of the polyhedra relies on the choice of parameter *a*: when *a* increases, more neighbours are included in each polyhedron, decreasing the number of holes in the overall hypervolume. However, a larger *a* also means a less precise volume around the points, including parts of the environmental space that are never encountered by the individual. The best value for this parameter can be chosen by visually assessing in 2D (for pairs of dimensions) the fit of the total hypervolume to the data points for different values of *a* (e.g. Fig S2).

#### Test of individual specialisation

The MISI provides a value of specialisation for each individual in the population. In order to test whether individuals are more specialised than expected by chance, a summary statistic of the central tendency of the MISI (e.g. the population median) can be compared with its null distribution via a recursive procedure of shuffling individual identity multiple times and recalculating the statistic. The most appropriate randomisation strategy will depend on the study system (population, season, stage, etc.), which can place particular constraints on individual movement and choices (e.g. seabirds are central-place foragers during the breeding season, and trip duration is restricted by the demands specific to incubation and chick-rearing duties).

By comparing the median of MISIs over the sampled population with the median for the same number of randomised individuals, it is possible to determine whether the population is composed of generalist or specialist individuals. If the empirical value of the median Multidimensional Individual Specialisation Index is higher than that expected by chance (95% CI of the null distribution), then the population can be considered made up of specialists (Fig. 2h); otherwise, it can be considered to constitute generalists. Individual MISIs can also be compared directly to contrast degrees of specialisation between individuals.

### Application to empirical data: albatross tracks

#### Tracking data

We used tracking data from grey-headed albatrosses (GHA) and black-browed albatrosses (BBA) breeding at Bird Island, South Georgia (54°00′S; 38°03′W), collected during the post-guard chick-rearing stage of the season. These data consist of locations obtained from Platform Terminal Transmitters (PTTs, see Phillips et al. 2004 for deployment details) for 124 trips of 5 male and 5 female GHA between February and March 2001, and 270 trips of 6 male and 6 female BBA between January and March 2002. Wet-dry (saltwater immersion) loggers were also deployed on BBA (Phalan et al. 2007), providing information on foraging activity. Locations were projected using the South Pole Lambert Azimuthal Equal Areas projection.

Locations in our datasets were at irregular time intervals (average interval ± SD of 1.3 ± 1.3 and 1.3 ± 1.0 h for GHA and BBA, respectively). To avoid the problem that the distribution of raw locations may not be representative of the actual time spent in each set of environmental conditions, locations were interpolated to hourly intervals using the package *adehabitatLT* (Calenge 2015) in R.3.4.2 (R Development Core Team 2017).

#### Environmental data

We selected variables reflecting oceanographic processes that are likely to affect individual choices in terms of location: sea surface temperature (SST), eddy kinetic energy (EKE), and depth. These variables have been shown previously to predict the distribution of albatrosses and other seabirds at the species level (see Wakefield et al. 2009a for a review, and Wakefield et al. 2011 for black-browed albatrosses in particular). For SST (in  °C), we used a weekly composite with a spatial resolution of 0.25° (NOAA Optimum Interpolation SST v2), obtained from the NOAA website (http://www.esrl.noaa.gov/). For EKE (in J), we used a weekly composite with a spatial resolution of 0.25°, downloaded from the AVISO website (www.aviso.oceanobs.com). For depth (in meters), we used a raster with a spatial resolution of 1′ downloaded from the NOAA website (www.ngdc.noaa.gov/mgg/global/). For each bird location, we extracted the value for SST and EKE on the closest date on which remote-sensing data were available, using the raster package (Hijmans and van Etten 2014) in R software.

We initially considered the inclusion of chlorophyll *a* concentration (Chl-a) in the models. However, this was discounted because our method requires an environmental estimate for each spatial position visited by the bird, and maps of Chl-*a* concentrations contained a high proportion of missing values at high latitudes due to cloud cover (57% missing values for BBA, 40% for GHA when using a weekly composite from the NOAA website: http://coastwatch.pfel.noaa.gov/).

#### Analyses

##### Selection of relevant locations

We removed transit locations based on a residence-time approach (Barraquand and Benhamou 2008), using the package *adehabitatLT* (Calenge 2015). This approach is based on the time spent in a circle of a certain radius around each focal point: locations around which individuals spend little time are interpreted as transit locations; conversely, locations around which individuals spent a lot of time are interpreted as foraging or resting locations (birds making sinuous movements or engaging in area-restricted search—ARS behaviour). First, we selected a radius of 45 km, obtained by multiplying the mean transit speed (which for BBA and GHA is 45 km/h, the best glide speed; Wakefield et al. 2009b) by the interval between sampling locations (1 h) (Torres et al. 2016). Second, in order to translate the residence times into categories of behaviour, we used the distribution of residence times (Fig. S1) to select a threshold value of 25,000 s (between the two peaks of the bimodal distribution). All locations corresponding to a shorter residence time were classified as transit, and all locations above that threshold were classified as foraging. To avoid using locations when birds might be drifting on the water (i.e. to separate resting from foraging), we considered only locations during daylight, when albatrosses are most likely to be foraging (Phalan et al. 2007). Timings of sunset and sunrise were calculated for each location using the *StreamMetabolism* package (Sefick Jr 2016) in R. As a validation of this approach to eliminate transit locations, we used wet-dry transitions (landings and take-offs) from immersion loggers, available only for BBA. As in Phalan et al. (2007), we characterised each 10-min bout as a “wet bout” if the bird spent more than 3 s on the water. Daytime locations were characterised as foraging if at least one bout in the surrounding hour (interval between two locations) was classified as wet. For each of these two approaches, we generated plots to visually inspect any differences, calculated the ISI values and tested their significance.

##### Individual foraging site fidelity

Specialisation in certain environmental conditions can be driven by several mechanisms, including specialisation in geographical space (i.e. “site fidelity”). The MISI method applied in geographical space provides information on the width of space use of each individual relative to that of the population. We thus calculated individual foraging site fidelity in this way for BBA and GHA. Note that as projected coordinates are in the same unit, they were not standardized prior to analysis.

##### Individual specialisation in environmental space

We assessed the level of individual environmental specialisation by applying the MISI approach to multi-dimensional environmental space (three-dimensions: SST, depth, EKE). We log-transformed EKE values to reduce the skew of the variables and standardised all variables (subtracting the mean and dividing the difference by the standard deviation across all individuals) to give the same weight to all dimensions. This minimises the influence of any single variable on the results. Flat polyhedra (arising when many points have similar coordinates in at least one dimension) led to computing problems with the R package *geometry* (Habel et al. 2015). This was avoided by adding a negligible random jittering (following a uniform distribution between − 5e-5 and + 5e-5) to each value.

##### Null model

**Fig. 3:**
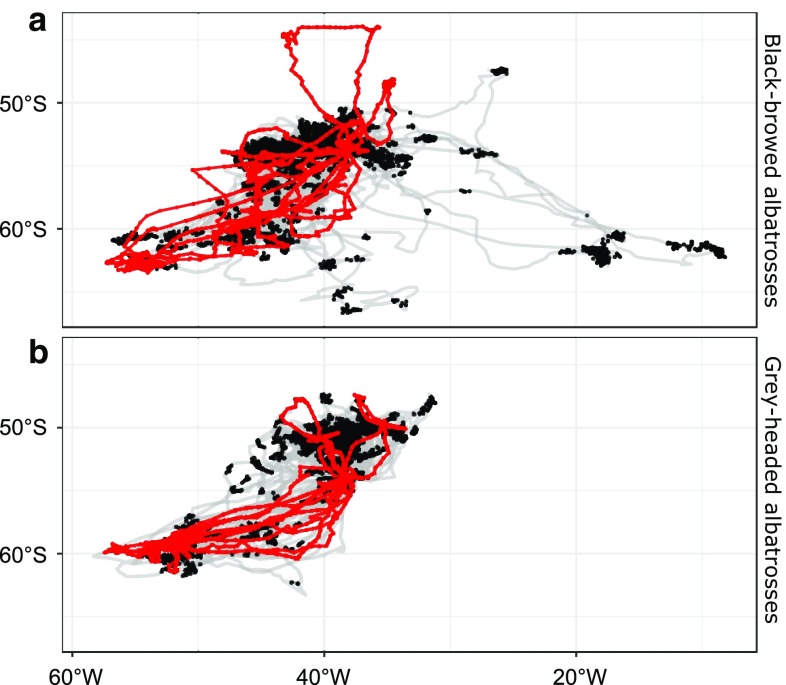
Examples of repeated trips by one individual contrasted with those of other individuals in the dataset: **a** BBA; **b** GHA. Grey: foraging trips of all sampled individuals. Red: foraging trips of one individual selected at random. Black dots: selected foraging locations used in the analysis

We built a null model for each species to test whether the MISI values obtained were different from the distribution expected by chance. To do so, we generated “null individuals” by randomly selecting subsets of all trips (e.g. Fig. 3) without replacement. Trips were assigned to individuals regardless of time (i.e. the order of trips was not kept) and of the sex of the individual (as these factors could not be taken into account given the sample size available. The number of trips per individual was drawn from the empirical distribution for that species. By keeping trips as whole units, we ensured that the null model took into account the spatial and temporal autocorrelation that exists within trips. We simulated 100 sets of as many null individuals as those in our dataset (12 for BBA and 10 for GHA) and calculated the MISI values for each of these sets, thus generating a null distribution of expected MISI values. We did not simulate a higher number of sets of null individuals due to computational limitations. We then compared the empirical MISI values with these distributions as a test of the extent to which they were significantly more specialised than expected by chance. For each species, and for each analysis (in geographic and in environmental space), the median of the empirical values for individuals was compared with the distribution of median values calculated for each null population (one median per set of 12 or 10 individuals). Significance of the one-way test (‘are MISI values higher than expected by chance?’) was assessed by calculating the proportion of null values higher than the empirical value.

## Results

### Data selection

**Fig. 4:**
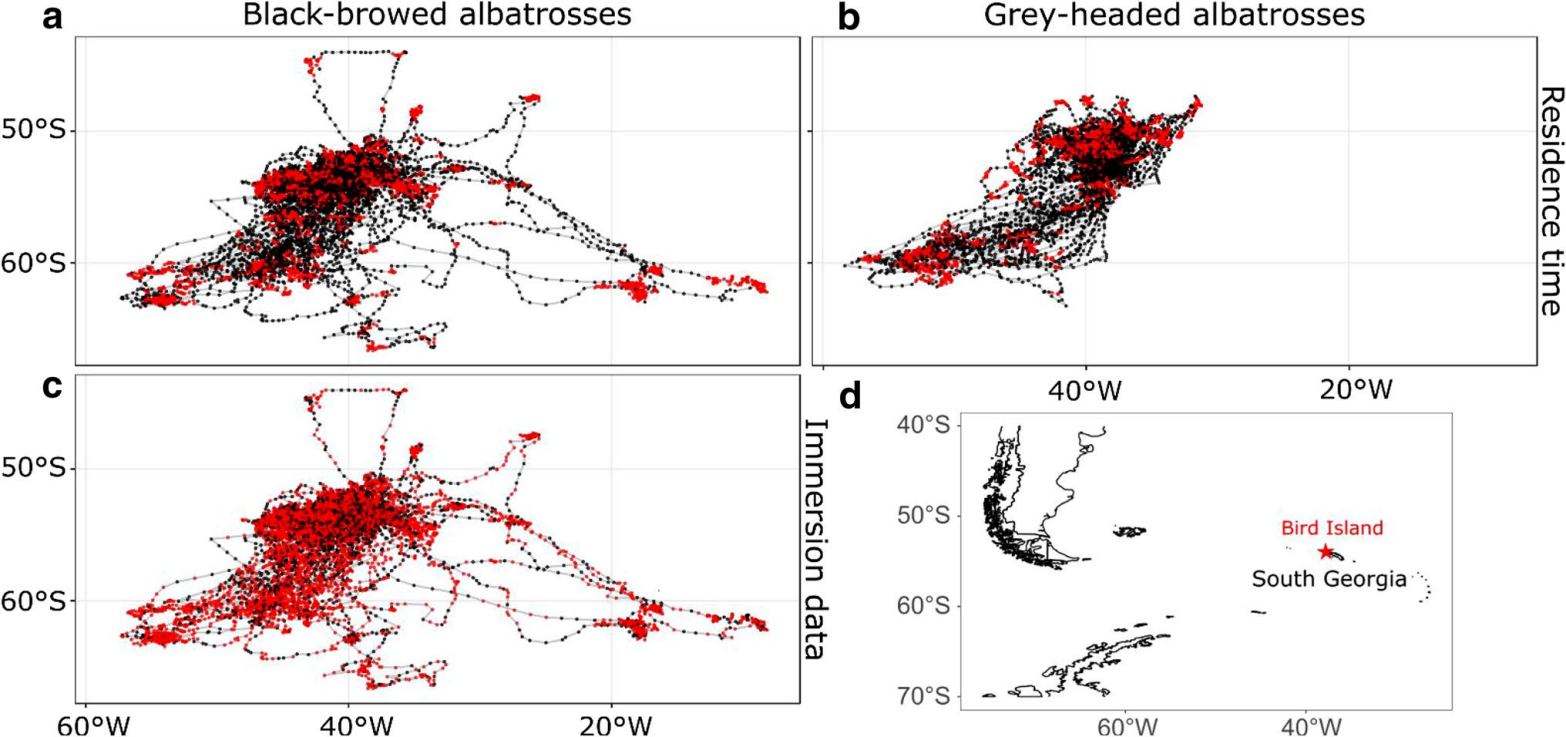
Locations where birds were considered to be foraging (red points) vs. in transit or resting (black points): **a** results for black-browed albatrosses (BBA) based on residence time, **b** results for grey-headed albatrosses (GHA) based on residence time, **c** results for BBA based on immersion data. Immersion data were not available for GHA. **d** Location of the colony (Bird Island, South Georgia)

**Fig. 5:**
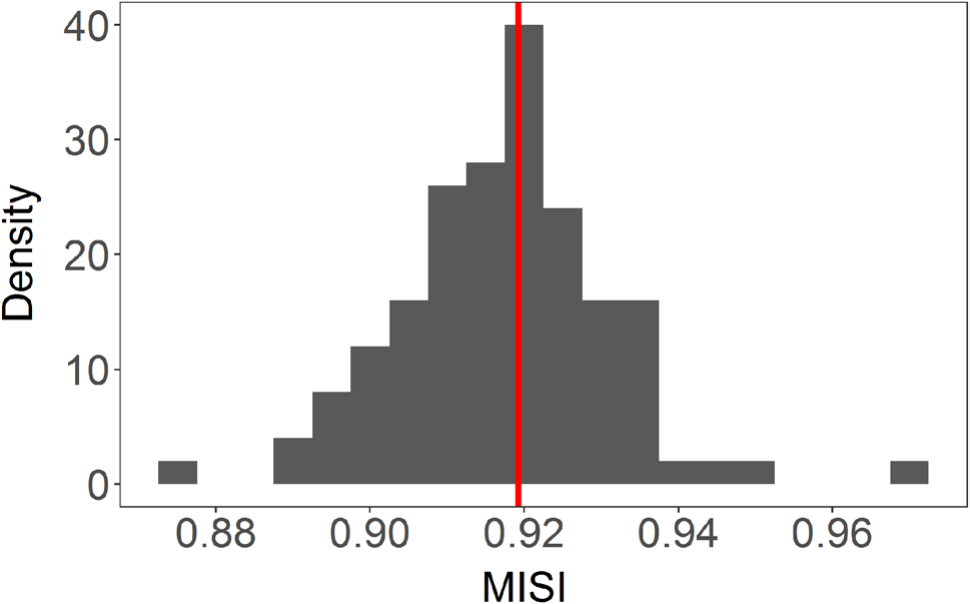
MISI values for BBA when locations were selected using immersion data: comparison between the null model (histogram; vertical blue dotted lines: 95% CI) and the empirical values (vertical red lines), in geographical space (site fidelity). Results are consistent with Fig. 6 when locations were instead selected using residence time

**Fig. 6:**
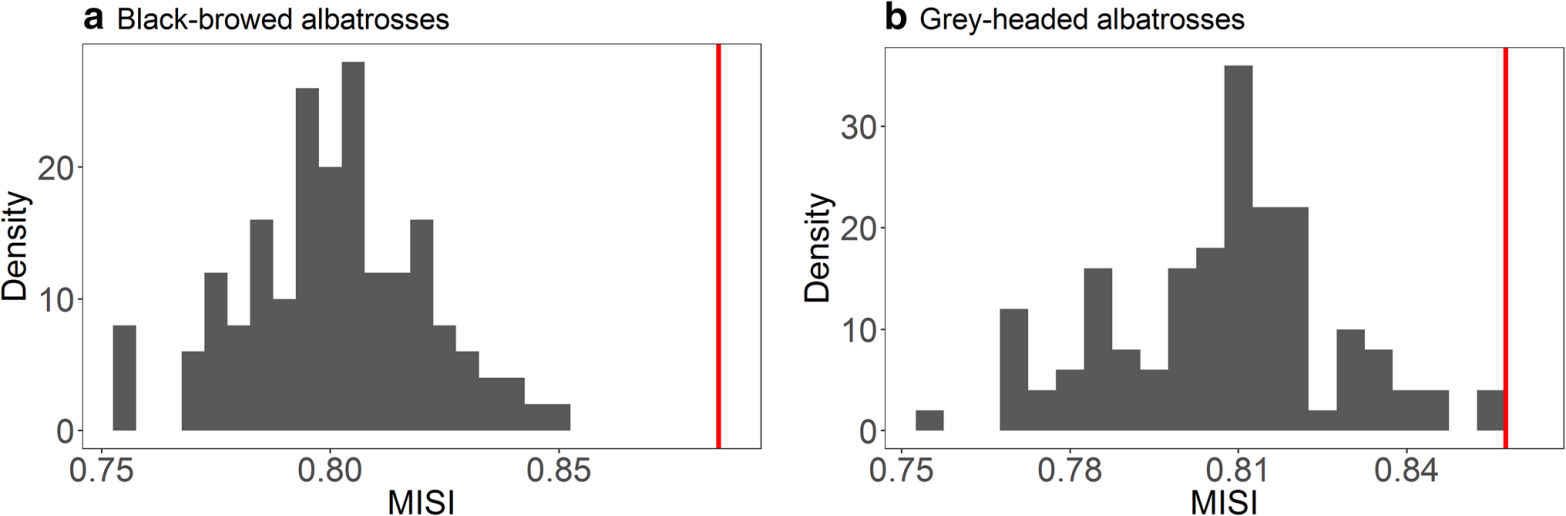
MISI values: comparison between the null model (histogram; vertical blue dotted lines: 95% CI) and the empirical values (vertical red lines), in geographical space (site fidelity). **a** Median population values for black-browed albatrosses (BBA); **b** median population values for grey—headed albatrosses (GHA). All birds were tracked during chick-rearing at South Georgia

Comparison between the two methods for classifying activity (residence time vs. immersion data) indicated that the areas inferred to be foraging locations were similar (Fig. 4). Moreover, the method used to identify foraging locations did not influence the conclusions of the MISI analysis (Figs. 5 and 6: in neither case was individual environmental specialisation detected for the BBA population). We were therefore confident that using presumed foraging locations selected using residence time was effective, and subsequent results are from these locations only. The final dataset in our MISI analyses included 1507 and 4243 locations, corresponding to 19 and 27% of the interpolated locations for GHA and BBA respectively (Fig. 4).

### Application of the MISI method: choice of parameters

In geographical space, when *a* was ≤ 200 km, some locations were not included in the hypervolumes, and the latter were too fragmented for further analysis (Fig. S2). At the other extreme, when *a* was ≥ 600 km, the hypervolumes included too many areas that did not contain data points (Fig. S2). We thus retained an intermediate adaptive-LoCoH parameter *a* = 400 km. In environmental space, we excluded values *a* < 2 because the hypervolumes were too fragmented and a > 4 because the hypervolumes covered too many areas with no data points (Figs. S3–5) and retained *a* = 3 for all subsequent analyses. Comparison between the results obtained for three different values within the realistic range of *a* in environmental space (*a *= 2, 3 and 4: Figs. S3–5) shows that the significance of the test was not affected by the value of *a*.

Selected grid cell sizes were 25 km and 0.125 in geographical and environmental space, respectively, which were a compromise between accuracy (Figs. S6 and S7) and computational time. Note that when variables are standardised (in environmental space), parameters (*a* and grid cell size) are unitless.

### Tests of individual specialisation

#### Individual foraging site fidelity

Both BBA and GHA showed evidence of significant individual site fidelity; empirical MISI values in geographical space were higher than expected by chance (*p* values < 0.01, Fig. 6). The effect was the strongest for BBA.

#### Individual environmental specialisation

**Fig. 7:**
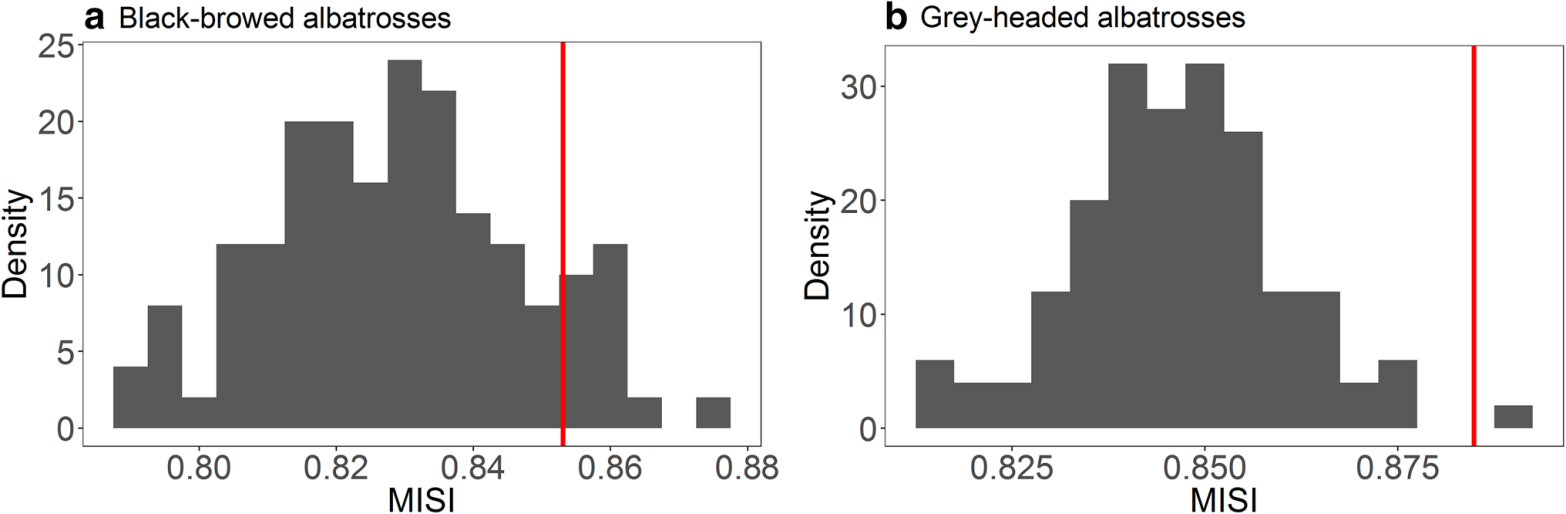
MISI values: comparison between the null model (histogram; vertical blue dotted lines: 95% CI) and the empirical values (vertical red lines), in three-dimensional environmental space (environmental specialisation). **a** Median population values for black-browed albatrosses (BBA); **b** median population values for grey-headed albatrosses (GHA)

There was no evidence of significant individual specialisation in three-dimensional environmental space (EKE, SST and depth) for BBA (*p* value = 0.12, Fig. 7). In contrast, individual GHA were significantly more specialised in three-dimensional environmental space than expected by chance (*p* value = 0.01, Fig. 7b).

## Discussion

### Ecological significance

In our study, both BBA and GHA were found to be site-faithful, although the effect was stronger for BBA. By comparison, only the GHA population appeared to be composed of individuals specialised in environmental space (habitat). We can be confident that the environment that is relevant for albatrosses was adequately described because our analysis included variables that were shown previously to influence habitat use at the species level in both BBA and GHA (Wakefield et al. 2011; Scales et al. 2016b). Hence, our results indicate that individual BBA do not differ in their habitat use with respect to the variables measured. This may be for two reasons: either individual birds use the same resources, or they specialise on different resources but these are not strongly linked to the environment. Indeed, even if resource specialisation exists, the scope for environmental specialisation to emerge depends on the level of predictability of the resource according to environmental cues (e.g. for site fidelity: Baylis et al. 2012; Wakefield et al. 2015). In particular, the higher the trophic level of a species, the more indirect the relationship between the environment and the distribution of its prey (Grémillet et al. 2008). The results found here for BBA diverge from those of Patrick and Weimerskirch for BBA from Kerguelen, which showed a stronger environmental specialisation (for water depth) than site fidelity, but are similar to those for northern gannets *Morus bassanus*, for which fidelity to the site is much stronger than that to the environment (Wakefield et al. 2015).

A possible explanation for the contrast between the results of Patrick and Weimerskirch (2017), who found evidence of individual specialisation in BBA from Kerguelen, and of our study in South Georgia, where we did not, may relate to the predictability of environmental conditions and resources in the surrounding waters. Prey availability in neritic habitats is considered to be more predictable than in the open ocean, and probably explains the high incidence of individual specialisation in near-shore species such as shags and cormorants (Phillips et al. 2017). BBA at Kerguelen forage far closer to the colony than adults from South Georgia, and spend a much higher proportion of their time in shelf waters (Fig. 2; cf. Weimerskirch et al. 1997; Pinaud and Weimerskirch 2002). Moreover, in a previous study comparing demography in the two populations, SST within the foraging range at Kerguelen was more stable between-years than at South Georgia (Nevoux et al. 2010). Other inequalities between the two sites could also influence the degree of specialisation, such as the greater diversity of available foraging habitats in the southwest Atlantic compared with the Indian Ocean (Phillips et al. 2009b). Alternatively, the differing results might relate to the relative constraint on foraging trip duration in each breeding stage. Our data were obtained during post-guard chick-rearing, whereas the previous study was during brood-guard (when the chick is attended by one parent, greatly limiting the time that the partner can spend at sea). However, in theory, greater constraints during brood-guard might reduce opportunities for specialising on different habitats. Adults in post-guard can target a wider range of habitats over a much larger area, which should reduce competition and hence the benefit of specialisation. Finally, the different results may be due to the different methodologies; the study at Kerguelen compared centroids of foraging areas (both in terms of geographic distance and differences in depth), which does not take into account the spread of the distribution (i.e. the variability in conditions used during each foraging phase). It also placed much less emphasis on the within-individual variance component, whereas our more robust comparison of niche volumes is likely to constitute a more conservative test of individual specialisation.

Our results were counter to our initial hypothesis, as we found stronger evidence for individual specialisation in foraging location in GHA than BBA. One explanation would be differences in predictability of environmental conditions and resources in foraging areas used by the study populations. During chick-rearing, GHA foraged both north of South Georgia, at the Antarctic Polar Front (APF), and over deep Antarctic waters, whereas BBA foraged mostly in open waters, or in shelf and shelf-slope waters locally or on the South Scotia Ridge, and very rarely at the APF (Fig. 4). This corresponds to the differences in diet between species in the study years: for GHA birds, diet in 2001 was dominated by cephalopods (~ 75 vs. only ~ 9% Antarctic krill *Euphausia superba*; British Antarctic Survey, unpublished data), whereas that for BBA diet was composed of krill (~ 52%) and fish (~ 30%). It might be that predictability of resources is higher than expected in areas visited by GHA. Indeed, the APF is a favoured foraging area throughout the year for this species (Xavier et al. 2003a, b; Clay et al. 2016), which suggests that the prey there are predictable to some extent. Additionally, although Antarctic krill are found in shelf, shelf-slope and deep waters around and to the south of South Georgia, there is huge spatial variation in their relative abundance, and concentrations are more ephemeral in the open ocean (Silk et al. 2016); hence the conditions experienced by BBA throughout their wide feeding distribution might be more unpredictable than expected.

### Measuring individual specialisation in multiple dimensions

In this paper, we present a method for quantifying individual site fidelity and consistency in habitat use from repeated observations of the same individuals, based on the hypervolumes of usage in a multidimensional space. Application of this approach in geographical space tests for site fidelity, and in environmental space for individual habitat specialisation. The basis of any study of individual differences is to contrast within-individual and between-individual variance components. Most studies of individual differences have used the repeatability framework (Nakagawa and Schielzeth 2010), where the between- and within-individual variance components are estimated via (Generalised) Linear (Mixed) Models. Repeatability is traditionally used in behavioural studies, for example in analyses of trip summary statistics (Patrick et al. 2014, Potier et al. 2015), such as path straightness, number of dives, bearing or maximum distance from the colony, but can also be used for studying habitat selection. In that case, environmental variables are included as fixed effects, and individual identities as random effects. This provides a separate measure of repeatability for each variable, but not a summarised output for each individual. More importantly, although interactions between variables can be included, the linear mixed-model approach does not directly take into account the multidimensionality of the niche. Here, we chose instead to extend the approach presented in Bolnick et al. (2003), which is a form of repeatability analysis but the within- and between-individual components are not calculated by estimating the parameters of linear models. Our novel formulation provides a single and readily interpretable index of specialisation for each individual, allowing for a robust statistical test of individual specialisation in situations when the environment is better characterized in multiple dimensions. The approach also presents various advantages over other methods for comparing distributions or habitat selection. For example, unlike measures of distance between centroids of distributions (both in geographical—e.g. Navarro and González-Solis 2009; Ceia et al. 2014, and in environmental space—e.g. Patrick and Weimerskirch 2017), our approach takes into account the volume of the niche. This is important as specialisation requires not only the mean of the distributions to differ between individuals, but also the spread of individual preferences to be narrow compared with the spread of preferences of the population as a whole.

To extend Bolnick’s approach to environmental conditions (habitat use), we had to estimate the within- and between-individual variances (and hence the total variance, to which they sum) in multiple dimensions. For that purpose, we built hypervolumes around points in a multidimensional space by generalizing the LoCoH method for estimating utilisation distributions in two dimensions (Getz et al. 2007). An alternative would have been to analyse parametric kernels, which are also used to estimate utilisation distributions in two dimensions. The R package hypervolume (Blonder et al. 2014) provides an approximation of these hypervolumes and their characteristics (e.g. volume). However, such parametric kernel methods often fail to capture features such as holes (but see Blonder 2016) or other sharp boundaries (Getz et al. 2007). Although more computationally intensive, the method proposed in our paper has the advantage of being able to deal with sharp boundaries, and of not extrapolating outside the environmental conditions of the most extreme locations, whatever the choice of the shape parameter.

Irrespective of the kernel method that is used, decisions need to be made concerning the parameters influencing the hypervolume construction. It is possible to exclude some values of the smoothing parameter (here, *a*) visually, either because too many points are excluded from the hypervolume, which contains too many holes (e.g. *a* = 200 km in Fig. S2), or because the hypervolume includes too many areas which do not contain points (e.g. *a* = 800 km in Fig. S2). Regardless, the test is robust to the choice of this parameter for a range of realistic *a* parameters (Fig. S8). The grain size of the grid used to estimate the volume of the hypervolumes also needs to be appropriate, but as demonstrated in this paper, it can be chosen empirically by increasing granularity until the volume estimates stabilise (Fig. S6–7).

### Considerations in the use of movement data for understanding the contribution of individuals to the niche of a species

Estimating site fidelity or individual environmental specialisation requires data from a series of repeated choices made by each individual. In particular, individual environmental specialisation can be studied by comparing environmental conditions (often derived from satellite remote-sensing data) in repeated choices of geographical locations to which animals are tracked on consecutive trips. In this study, we used PTT data for two species of central-place foragers which made multiple trips from their colony during chick-rearing. Given that within each trajectory there is a degree of spatial autocorrelation (because once a decision is made to travel in a particular direction, the options are then restricted to the environment and areas available in the surroundings), we considered the foraging trip as our unit of study. Our method thus requires having a sufficient number of tracks per individuals. We have assumed that each foraging trip constitutes an independent choice of foraging areas, but there are limitations to this assumption. First, if the study is based only on portions of tracks (in this application: foraging trips) that are consecutive, there is a risk that individuals return to a location because they successfully obtained prey there on the previous trip (win-stay lose-shift strategy), and not because of long-term site fidelity or environmental specialisation (but see Wakefield et al. 2015 for a discussion on how these can be disentangled). It is thus important to use a time series of tracks that is long enough that the decisions can be considered sufficiently independent, with the limitation that periods in which behaviour may change markedly are analysed separately (e.g. habitat selection often differs between the breeding and non-breeding seasons, because the central-place spatial constraint is removed and thus the availability of environmental conditions may differ markedly; Phillips et al. 2017). Second, even if portions of tracks can be considered independent from one another, they do not necessarily constitute distinct choices. For example seabirds, and in particular the two species of albatrosses studied here, can be strongly constrained by wind (Weimerskirch et al. 2000), which influences their spatial distributions (Phillips et al. 2004; Weimerskirch et al. 2012). Failing to account for such wind effects might be misleading: for instance, two birds leaving the colony on the same day might make similar trips not because they have similar environmental preference but because they experience the same wind conditions. Also, an individual might not return to the same place from one trip to the next, not because the place is unsuitable, but because the wind conditions would make this inefficient. However, in our study, we removed transit locations to focus on foraging areas: hence, although wind can influence the general direction (and thus the identification of site fidelity), it should be less of an issue for environmental specialisation, because of the redundancy of environmental conditions in geographical space (i.e. the same environment can be found in different places, and albatrosses cover great distances whilst foraging).

### Characterising the niche: strength of environmental drivers and accessibility

The relevance of any analysis of individual specialisation in environmental space relies on the strength of the relationship between the species under study and its environment. This relationship is both related to how strongly the environment affects the distribution of the species (i.e. the strength of the biological constraints), and the extent to which the environmental variables that matter have been incorporated in the study (which depends on available data, as well as on the analyst’s capacity to identify the important habitat variables at the appropriate scale). Thus, failure to detect individual specialisation can either mean that it does not exist, or that the environment has been incorrectly characterised (either because some important environmental variables were neglected, or measured at the wrong scale; Scales et al. 2016a).

Additionally, and contrary to model-based approaches (e.g. resource selection functions), our method requires all individuals to have access to the same environmental conditions, so that comparing choices of foraging locations between individuals is equivalent to comparing environmental preferences. Our method is thus particularly suited for studying individual specialisation in central-place foragers, even though they are more constrained in their choices of foraging locations (reducing the scope for specialising). Furthermore, although the notion of accessibility also depends on the scale of study (once the first decision about direction is made, the available foraging areas are no longer the same for all individuals), the redundancy of environmental conditions in space would allow specialised individuals to access patches of their preferred habitat even in a different foraging area.

### Conservation implications

Tracking data are used increasingly to help identify key foraging sites that could be included in a network of marine protected areas (Lascelles et al. 2016; Tancell et al. 2016), or for understanding potential spatial overlap with fisheries (Phillips et al. 2005; Pichegru et al. 2009; Zydelis et al. 2011). However, resources for research are limited, and our results have implications for how these might be best-targeted. If all individual animals behave in a similar way (i.e. are generalists), then effort should be focused on a thorough understanding of what each individual does, i.e., it is better to track fewer individuals for a long period of time. On the contrary, if most animals in the population are specialists, efforts should be dedicated to tracking as many individuals as possible, even if for shorter periods. This holds not only with regard to specialisation in foraging areas (i.e. site fidelity), but also environmental preferences. Indeed, understanding the environment selected by individuals provides information on the processes and mechanisms driving geographic distributions, allows the integration of dynamic variables, and is thus useful to predicting distributions based on future environmental conditions (e.g. climate change, or in relation to seasonal and annual variation). Besides, as there is some degree of redundancy of environmental conditions in geographical space, fewer individuals are needed to characterise all the environments than the locations (areas) used by the population. Nevertheless, although our results indicate that the balance should be towards intensive rather than extensive sampling of individuals, this needs to be nuanced by the possibility that unsampled individuals with a different strategy might be present in the population.

### Potential for further applications of the method

The framework presented here offers the advantage over previous methods in that it provides a value for the level of individual specialisation of each individual within the population, thus allowing ranking and tests for the short- or long-term consequences of such specialisation. This contrasts with previous studies, which usually compare groups of specialists and generalists (Phillips et al. 2017). Hence our approach can be adopted in short-term studies investigating the relationship between degree of individual specialisation and foraging efficiency, and long-term studies which link it to breeding success and carry-over effects (e.g. in Patrick and Weimerskirch 2017).

Our method can also be used to test hypothesis regarding the relationship between individual specialisation and individual life-history traits (e.g. age, sex, personality). For example, juveniles and adults from the same colony can be compared, providing insights into the learning processes driving foraging site selection. We expect younger individuals to be less specialised than adults, and the onset of greater specialisation to indicate when learning occurs. Differences in movement capacities between juveniles and adults have been shown in several species, including wandering albatrosses (*Diomedea exulans*) (Riotte-Lambert and Weimerskirch 2013, de Grissac et al. 2016) and Scopoli’s shearwaters *Calonectris diomodea* (Péron and Grémillet 2013). Increases in site fidelity with age have also been recorded (in Weddell seals *Leptonychotes weddellii*, Cameron et al. 2007, in sanderlings *Calidris alba*, Lourenço et al. 2016, and in northern gannets Votier et al. 2017), but to our knowledge, there has been no study comparing the degree of environmental specialisation between adults and juveniles. Patrick and Weimerskirch (2017) found no effect of age on the degree of site fidelity or individual specialisation, but they only studied adults which will have largely passed through the learning period.

Finally, our framework can also be used to investigate other ecological questions, such as the influence of trophic level on the degree of individual specialisation in different species or the influence of the environment on the emergence of individual specialisation (e.g. depending on the level of resources, and so on the level of intra-specific competition, the advantages of specialism will change). Providing the habitat can be properly described and enough (independent) data are available for each individual, it would be informative to apply this method to a wide range of taxa to understand more aspects of the individual component of niches.

## Electronic supplementary material

Below is the link to the electronic supplementary material.

Supplementary material 1 (TXT 1 kb)

Supplementary material 2 (CSV 606 kb)

Supplementary material 3 (R 4 kb)

Supplementary material 4 (R 4 kb)

Supplementary material 5 (R 15 kb)

Supplementary material 6 (DOCX 766 kb)
