# Supplementary material for: Quantifying individual specialization using tracking data: a case study on two species of albatrosses
Source: Mar Biol. 2018 Sep 8;165(10):152. doi: 10.1007/s00227-018-3408-x (PMC6132544; doi:10.1007/s00227-018-3408-x)
Supplement: Supplementary file 6 — Supplementary material 6 (DOCX 766 kb) [file 227_2018_3408_MOESM6_ESM.docx]

# Appendices

## Appendix S1


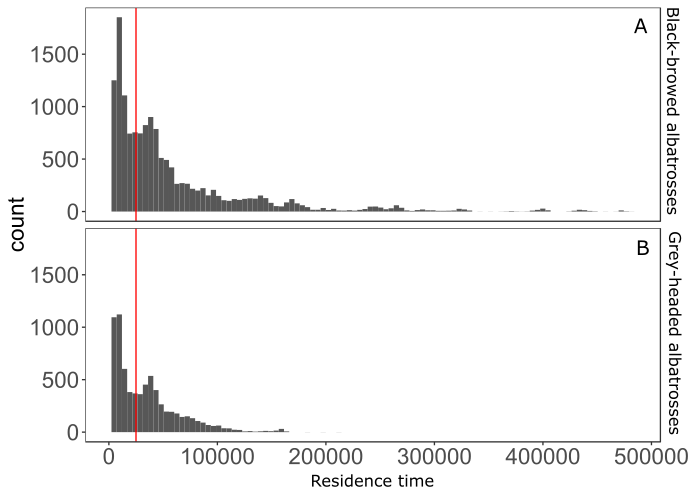


Figure S1: Distribution of residence times for A) black-browed albatrosses, B) grey-headed albatrosses tracked from South Georgia during chick-rearing. Vertical red line: selected threshold value for the selection of locations for the analyses.


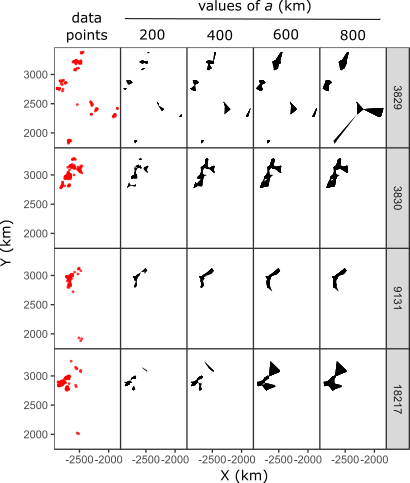


Figure S2: Effects of different values for parameter a on the resulting hypervolumes in geographical space for black-browed albatrosses.


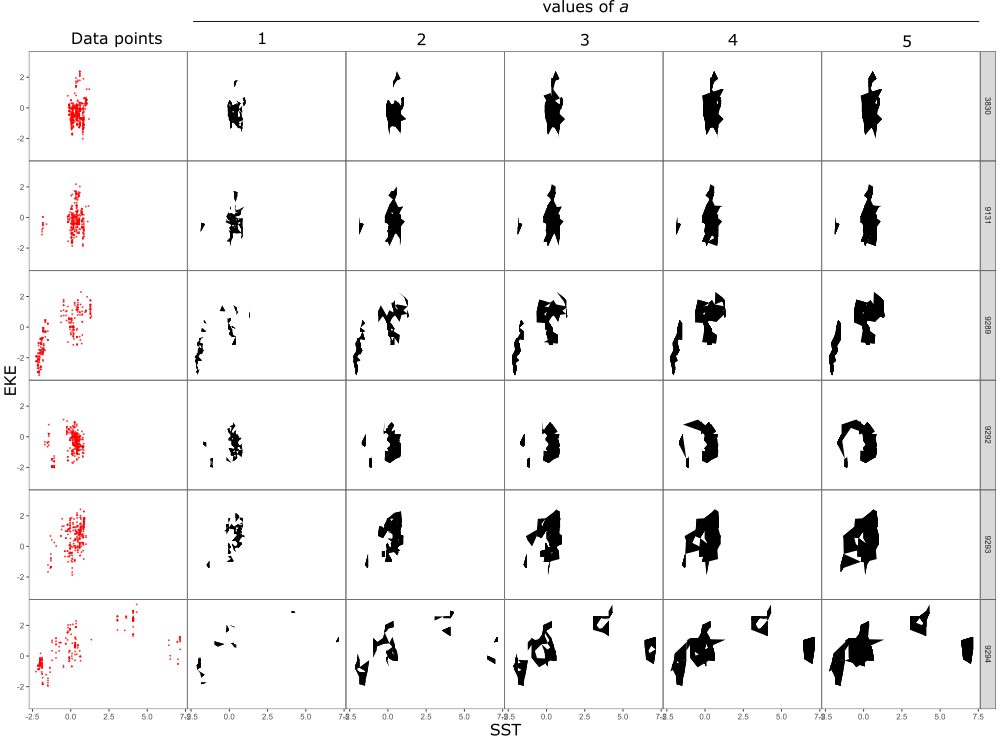


Figure S3: Effects of different values for parameter a on the resulting hypervolumes in environmental space for black-browed albatrosses, projecting in the EKE-SST plane.


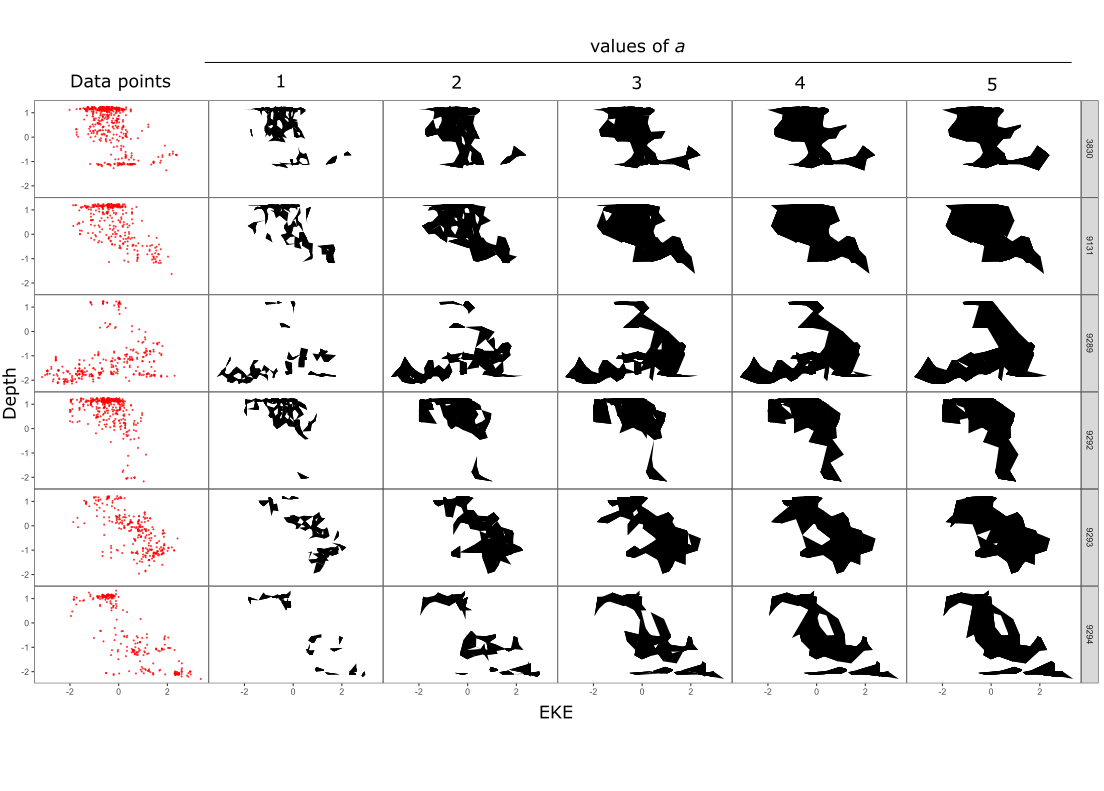


Figure S4: Effects of different values for parameter a on the resulting hypervolumes in environmental space for black-browed albatrosses, projecting in the EKE-depth plane.


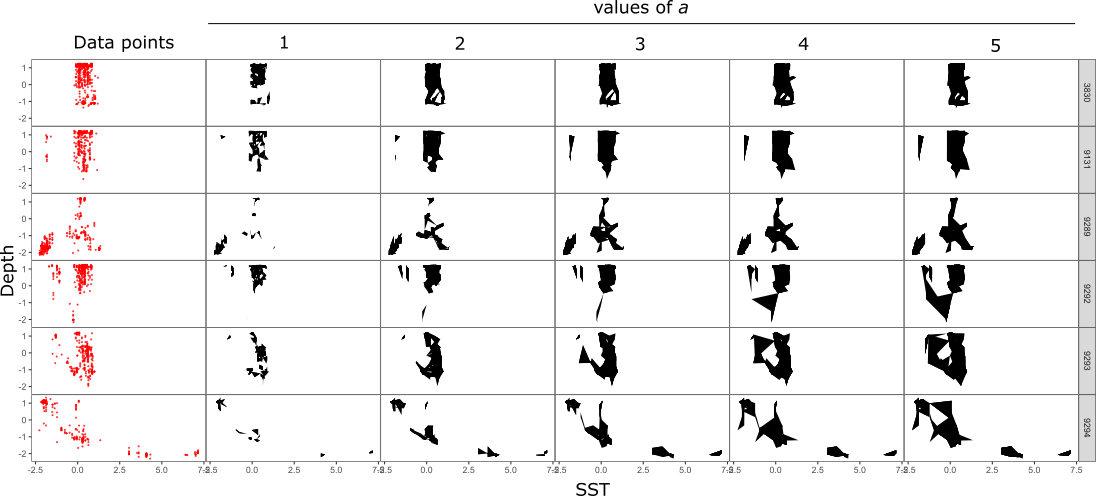


Figure S5: Effects of different values for parameter a on the resulting hypervolumes in environmental space for black-browed albatrosses, projecting in the SST-depth plane.


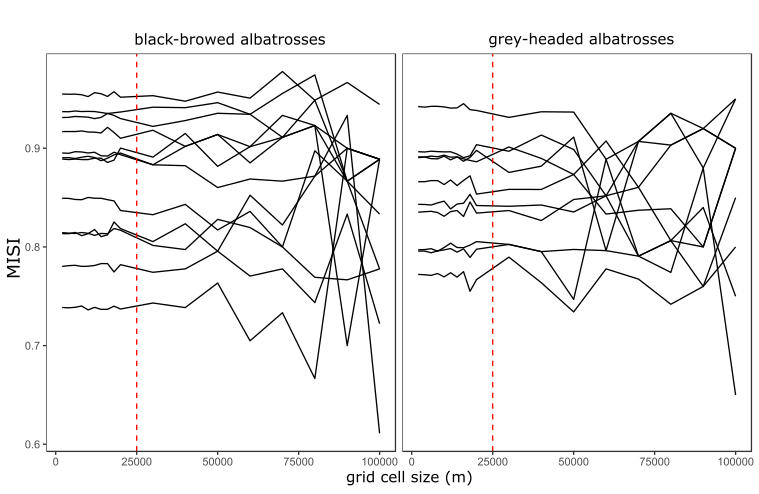


Figure S6: Effect of different grid cell sizes on the resulting multidimensional individual specialisation index (MISI) in geographical space. Red dotted line: grid cell size used for the analysis.


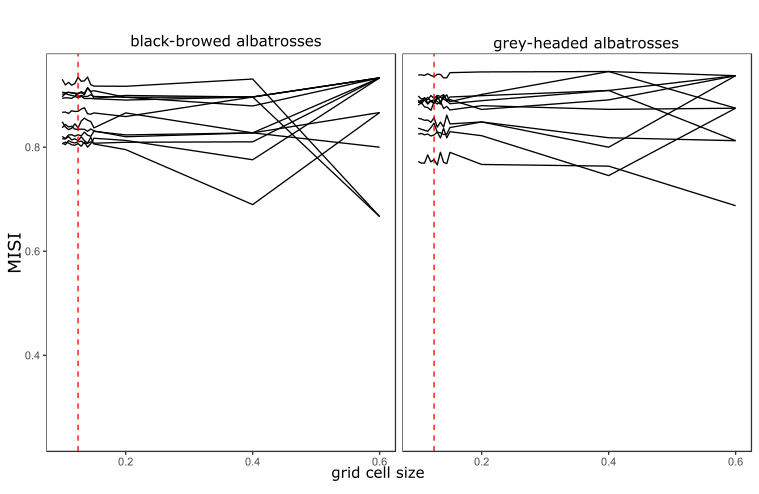


Figure S7: Effect of different grid cell sizes on the resulting multidimensional individual specialisation index (MISI) in environmental space. Red dotted line: grid cell size used for the analysis.


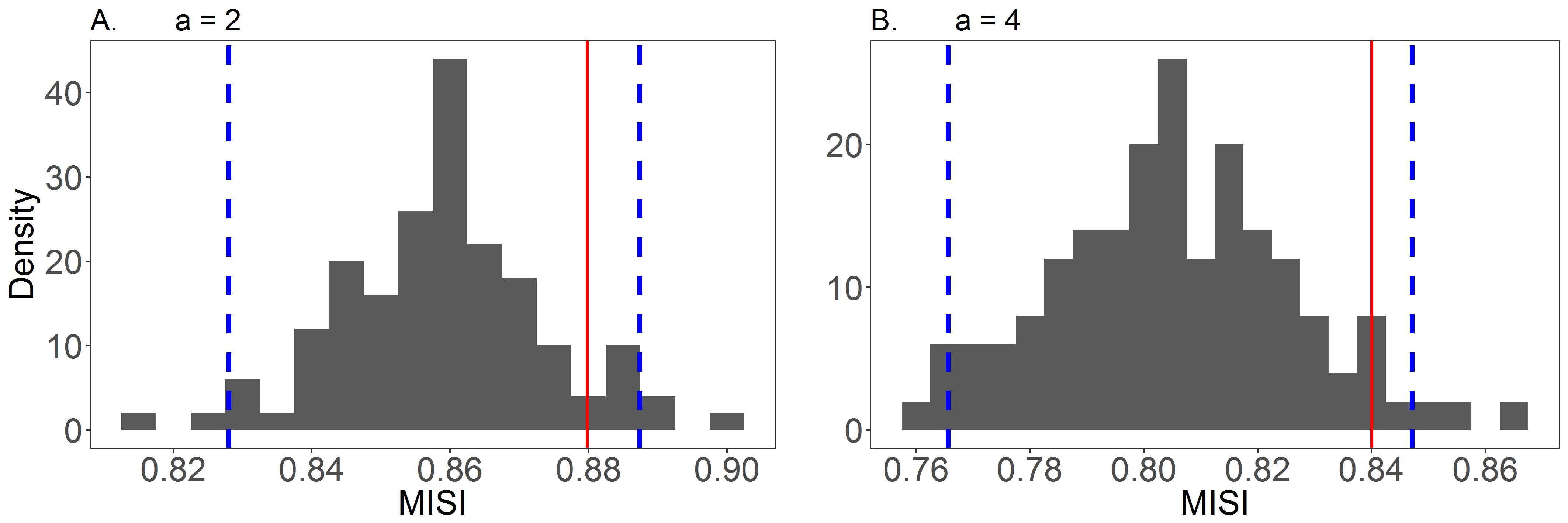


Figure S8: Effect of values for parameter a on ISI values: comparison between the null model (histogram; vertical blue dotted lines: 95% CI) and the empirical values (vertical red lines), in three-dimensional environmental space for black-browed albatrosses: A) median population values for a = 2; B) median population values for a = 4.


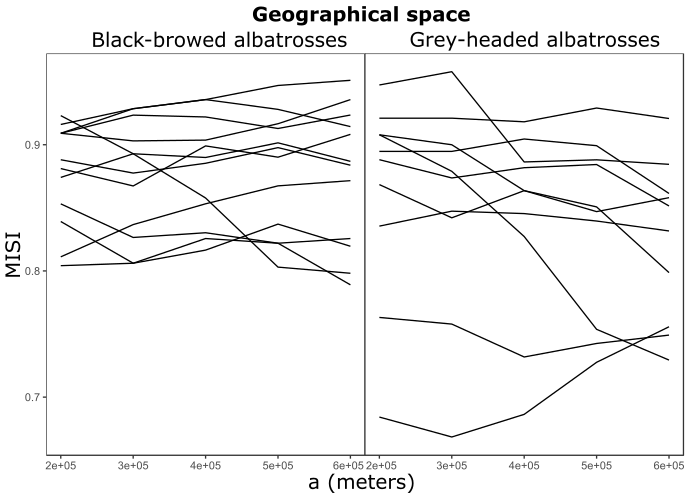


Figure S9: Effect of different values of *a* (smoothing parameter) on the resulting multi-dimensional individual specialisation index (MISI) in geographical space.


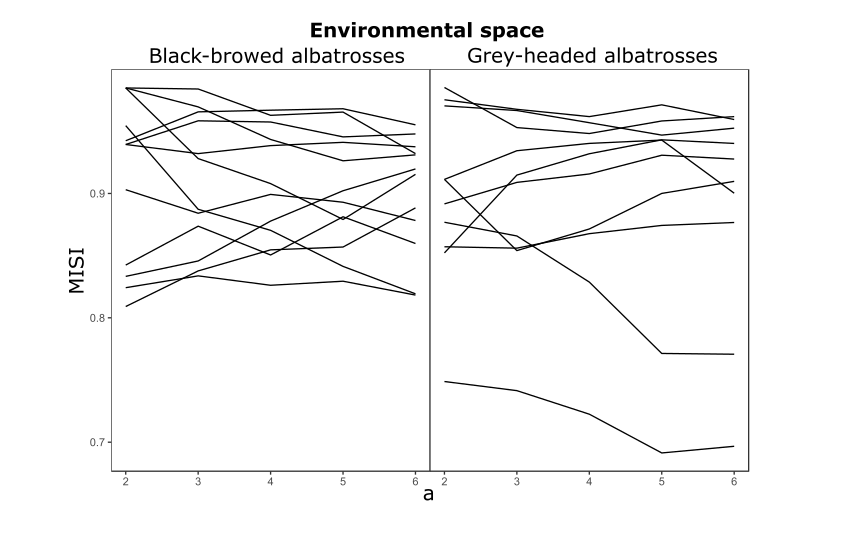


Figure S10: Effect of different values of a (smoothing parameter) on the resulting multi-dimensional individual specialisation index (MISI) in environmental space.
